# Reduction in mucosal‐associated invariant T cells (MAIT) in APECED patients is associated with elevated serum IFN‐γ concentration

**DOI:** 10.1002/eji.202451189

**Published:** 2024-09-18

**Authors:** Iivo Hetemäki, Joona Sarkkinen, Huai Hui Wong, Nelli Heikkilä, Saila Laakso, Simo Miettinen, Mikko I. Mäyränpää, Outi Mäkitie, T Petteri Arstila, Eliisa Kekäläinen

**Affiliations:** ^1^ Translational Immunology Research Program University of Helsinki and Helsinki University Hospital Helsinki Finland; ^2^ ImmuDocs National Doctoral Education Pilot Program University of Helsinki Helsinki Finland; ^3^ Children's Hospital University of Helsinki and Helsinki University Hospital Helsinki Finland; ^4^ Research Program for Clinical and Molecular Metabolism, Faculty of Medicine University of Helsinki Helsinki Finland; ^5^ Folkhälsan Research Center Institute of Genetics Helsinki Finland; ^6^ Department of Pathology University of Helsinki and Helsinki University Hospital Helsinki Finland; ^7^ Department of Molecular Medicine, Karolinska Institutet, and Clinical Genetics Karolinska University Hospital Stockholm Sweden

**Keywords:** AIRE, Anti‐cytokine antibodies, APECED, APS‐1, IFN‐γ, IL‐18, Inborn errors of immunity, MAIT

## Abstract

Mucosal‐associated invariant T cells (MAIT) are innate‐like lymphocytes enriched in mucosal organs where they contribute to antimicrobial defense. APECED is an inborn error of immunity characterized by immune dysregulation and chronic mucocutaneous candidiasis. Reduction in the frequency of circulating MAITs has been reported in many inborn errors of immunity, but only in a few of them, the functional competence of MAITs has been assessed. Here, we show in a cohort of 24 patients with APECED, that the proportion of circulating MAITs was reduced compared with healthy age and sex‐matched controls (1.1% vs. 2.6% of CD3^+^ T cells; *p* < 0.001) and the MAIT cell immunophenotype was more activated. Functionally the IFN‐γ secretion of patient MAITs after stimulation was comparable to healthy controls. We observed in the patients elevated serum IFN‐γ (46.0 vs. 21.1 pg/mL; *p* = 0.01) and IL‐18 (42.6 vs. 13.7 pg/mL; *p* < 0.001) concentrations. Lower MAIT proportion did not associate with the levels of neutralizing anti‐IL‐22 or anti‐IL‐12/23 antibodies but had a clear negative correlation with serum concentrations of IFN‐γ, IL‐18, and protein C‐reactive protein. Our data suggest that reduction of circulating MAITs in patients with APECED correlates with chronic type 1 inflammation but the remaining MAITs are functionally competent.

## Graphical abstract

We found a reduced frequency of circulating mucosal‐associated invariant T cells (MAITs) in patients with APECED with a clear negative correlation to inflammatory markers CRP, IL‐18, and IFN‐γ. These findings suggest that the decrease in MAITs is secondary to type 1 immune dysregulation present in the disease.

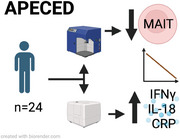

## Introduction

Human inborn errors of immunity (IEI) have revealed important pathways governing mucosal immunity. One of these diseases is an inborn immune dysregulation syndrome called autoimmune polyendocrinopathy, candidiasis, and ectodermal dystrophy (APECED) resulting from mutations in the autoimmune regulator (AIRE). AIRE regulates the expression of tissue‐restricted antigens in thymic medullary epithelial cells [1]. Lack of AIRE leads to failure of negative selection and escape of autoreactive T lymphocytes to the periphery. True to its name, APECED is characterized by autoimmunity against especially endocrine organs and mucocutaneous candidiasis [2]. A key feature of the disease is the almost uniform production of neutralizing anti‐cytokine antibodies toward type I interferons that predispose the patients to severe viral infections [3, 4].

A large proportion of APECED patients have also antibodies against Th17 cytokines IL‐17 which are important in mounting effective anti‐fungal immune responses, and patient T cells have a lower production of IL‐22 after microbial stimulation [5, 6]. Hence, predisposition to mucocutaneous candidiasis in APECED has been suggested to be an additional autoimmune manifestation attributed to the anti‐cytokine antibodies causing a defective Th17 response against *Candida* spp. A recent analysis of oral mucosal immunity both in patients with APECED and in Aire^−/−^ mice challenged this theory, as the authors could not detect a mucosal Th17 defect, but instead demonstrated that the infiltration of IFN‐γ producing CD4^+^ and CD8^+^ T cells into the oral mucosa impaired the mucosal integrity and predisposed to oral candidiasis [7]. We have since replicated these findings in samples from genital mucosa [8]. Dysregulated skewing of mucosal effector cells toward type I inflammation, potentially evoked by the absence of an efficient Th17 response, could therefore be a contributing factor in chronic mucocutaneous candidiasis. In all, the underlying cause of the increased susceptibility to *Candida* spp. infection in APECED remains unresolved and is most likely multifactorial.

Tissue‐resident and innate‐like lymphocytes are key effectors in the mucosal immune system. Mucosal‐associated invariant T cells (MAIT) are innate‐like lymphocytes enriched in the mucosal tissues while in circulation their frequency is 1 to 10% of αβ T cells [9]. MAITs are characterized by expression of the semi‐invariant T‐cell receptor, consisting of Vα7.2 paired with a limited repertoire of Vbeta chains, and high expression of CD161 (KLBR1, killer cell lectin‐like receptor subfamily B, member 1) which is typically expressed by innate lymphocytes, such as natural killer cells. The best‐defined role for the semi‐invariant MAIT TCR specific for MAITs is to recognize microbe‐originating riboflavin (B2 vitamin) metabolites presented in the major histocompatibility complex class 1‐related protein (MR1) [10]. MAITs can also be activated without TCR involvement by inflammatory cytokines alone, especially IL‐18 [11, 12, 13]. MAITs are believed to contribute to antimicrobial defense at mucosal sites by producing cytokines and performing cytotoxic effector functions but their role in autoimmunity is still unclear since both pathogenic and protective roles have been proposed [14]. Preliminary support for MAIT cell involvement in APECED came from a report where a reduced frequency of circulating MAITs was described in a cohort of eight patients [15].

Reduction in the frequency of MAITs is not an uncommon finding in inborn errors of immunity, but apart from a few conditions (such as MR1 deficiency), a direct causal link between the genetic defect and reduction in MAITs has not been established [16]. Here, we carried out a comprehensive immunophenotypic and functional characterization of MAITs in a large Finnish cohort of patients with APECED. We show that a reduction of circulating MAITs in patients with APECED correlated with chronic type 1 inflammation while the remaining MAITs appeared functionally competent.

## Results and discussion

### Patients with APECED have a decreased proportion of circulating MAITs

**Figure 1:**
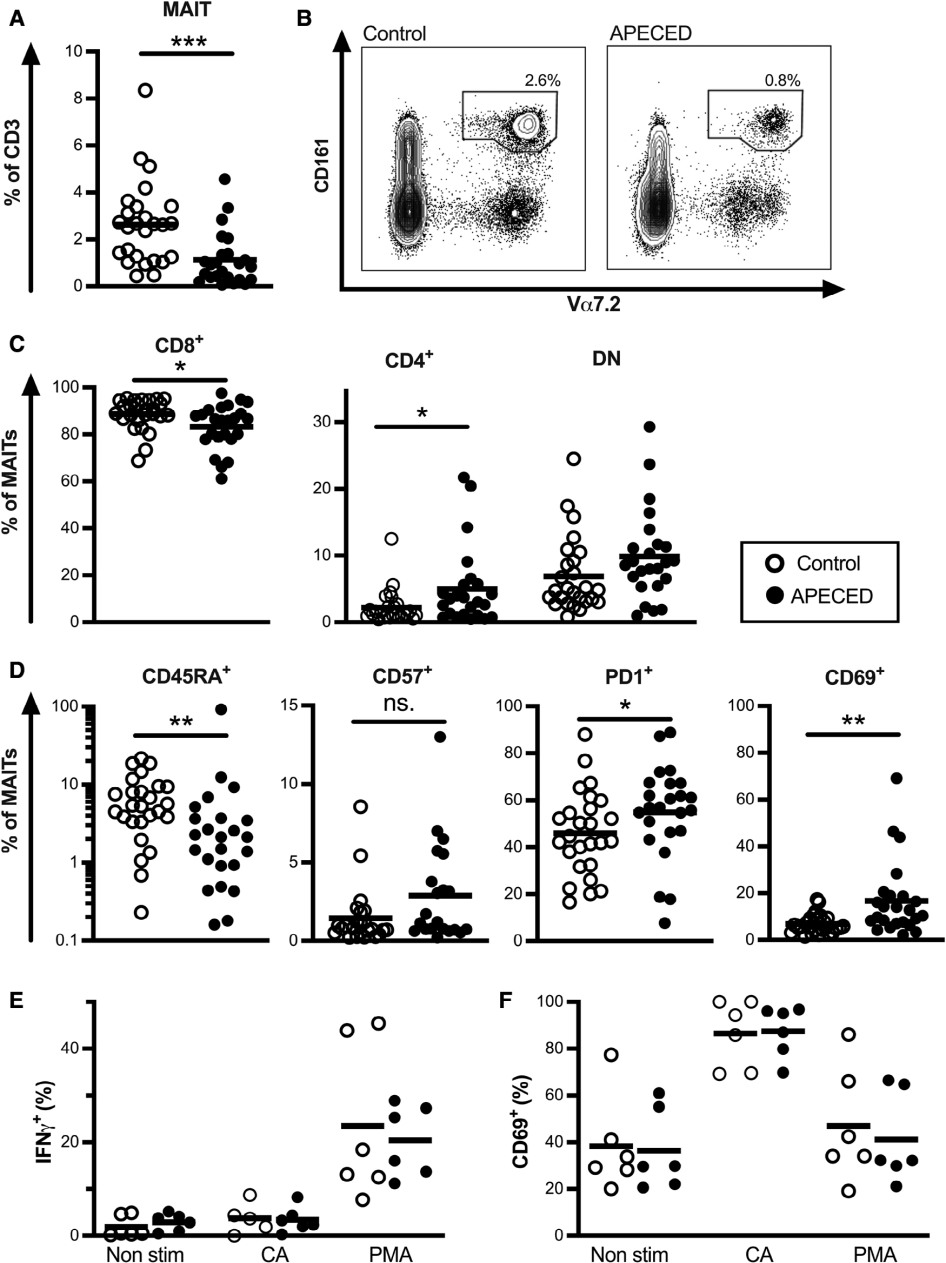
Frequency and function of MAITs in patients with APECED. (A) Frequency of blood CD3^+^CD161^++^Vα7.2 MAITs in controls (open circle) and patients with APECED (black circle). (B) A representative flow cytometry plot for MAIT cell frequency of CD3^+^ T cells for control and a patient is shown. (C) Frequency of CD4^−^CD8^+^, CD4^+^CD8^−^, and CD4^−^CD8^−^ double negative (DN) cells of MAITs in controls and patients. (D) Frequency of CD45RA^+^, CD57^+^, PD1^+^, and CD69^+^ cells of MAITs in controls and patients. (E) Frequency of IFN‐γ^+^ and (F) CD69^+^ MAITs without stimulation, after stimulation with *C. albicans* (CA), and after stimulation with PMA in controls and patients. The gating strategy for (A–D) is shown in Supporting Information Fig. S1A and for (E and F) in Supporting Information Fig. S2. Statistical significance was calculated with Mann–Whitney *U*‐test; *p < 0.05, **p < 0.01, ***p < 0.001, ns. = non‐significant.

We assessed the frequency of circulating MAITs in peripheral blood in 24 patients with APECED at the mean age of 40.4 years (age range 7–70; 13 females). The relative abundance of CD3^+^Vα7.2^+^CD161^++^ MAITs was markedly reduced in patients compared with age and sex‐matched healthy controls (*n* = 26) (1.1% vs. 2.6% of CD3^+^ T cells; Mann–Whitney *U* test, *p* < 0.001; Fig. 1A and B). Most MAITs are CD8^+^ and this population is the most well‐studied, while the role of CD8^−^CD4^−^ double negative (DN) and CD4^+^ MAITs remains more elusive. DN MAITs have been suggested to be an MAIT subset arising after prolonged TCR‐mediated stimulation [17]. In patients with APECED, we saw a reduction in the CD4^−^CD8^+^ MAIT subset compared with controls (83.2 vs. 88.7% of MAITs; *p* = 0.02; Fig. 1C) with a relative increase in CD4^+^CD8^−^ cells (5.0 vs. 2.2% of MAITs; *p* = 0.05; Fig. 1C) and no difference in DN MAIT subset (9.9 vs. 6.9% of MAITs; ns.).

While the TCR Vα7.2^+^ CD161^++^ population is enriched for MAITs, it also contains conventional T cells. To confirm our findings, we used MR1 tetramer loaded with 5‐(2‐oxopropylideneamino)‐6‐D‐ribitylaminouracil (5‐OP‐RU) that is specific for the invariant TCR found in the majority of MAITs [18]. A vast majority of CD3^+^Vα7.2^+^CD161^++^ cells stained positive with MR1 tetramer in both patients and healthy controls (94.9 vs. 95.0%, ns.; *n* = 4+7; Supporting Information Fig. S1C). In CD4^+^ subset of CD3^+^Vα7.2^+^CD161^++^, there was considerable individual variation in the fraction of cells staining positive for MR1‐5‐OP‐RU tetramer in both patients and controls (range 9–76%; Supporting Information Fig. S1C). The decrease in the CD8^+^ subset with a reciprocal increase in CD4^+^ and DN subsets was observed also in the MR1‐5‐OP‐RU tetramer positive MAITs (Supporting Information Fig. S1D). However, using this gating strategy the frequency of CD4^+^ MAITs was lower in both patients and controls than with Vα7.2^+^ CD161^++^ gating reflecting variable contamination of conventional CD4^+^ T cells to the latter population. This contamination might be proportionally higher in APECED due to a general decrease in circulating MAITs.

MAIT cell depletion has been associated with exhaustion and excessive activation in several chronic viral infections [14]. We therefore next examined MAIT maturation and activation status in APECED patients. In patients, a higher percentage of MAITs were CD45RA‐ memory phenotype than in controls (89.1 vs. 84.6% of MAITs; *p* = 0.002; Fig. 1D). One patient was a clear outlier with 98% of MAITs being CD45RA^+^. Expression of CD57, a marker of T cell senescence, was comparable in patients and controls (3.1 vs. 2.4% of MAITs; ns.; Fig. 1D) while expression of exhaustion marker PD‐1 was higher in patients compared with healthy controls (54.9 vs. 46.0; *p* = 0.04; Fig. 1D). Finally, the patients’ MAITs displayed markedly higher levels of activation and tissue retention marker CD69 (16.7 vs. 6.8%; *p* = 0.002; Fig. 1D).

Next, we stimulated peripheral blood mononuclear cells (PBMCs) with PMA/ionomycin to evaluate the ability of MAITs to produce cytokines. To evaluate activation via the MR1 — invariant TCR route we used inactivated *C. albicans* to stimulate MAITs in vitro. PMA stimulation resulted in good IFN‐γ^+^ production while with *C. albicans* stimulation IFN‐γ^+^ production was less pronounced despite robust upregulation of CD69. We did not see any difference in response between patients and controls (*n* = 6+6) to either of the stimulations (Fig. 1E and F). We also analyzed expression of chemokine receptors CCR2, CCR4, CCR5, CCR6, CCR7, CXCR3, and CXCR5 ex vivo in MAITs (*n* = 6+6). Almost all MAITs expressed CCR2 and CCR5 in both patients and healthy controls reflecting their highly inflammatory nature, also CCR6 expression was high in both groups (Supporting Information Fig. S3). We detected no difference in the expression of chemokine receptors between the groups.

In conclusion, in a cohort of 24 APECED patients representing approximately one‐third of living Finnish patients with APECED, we found a clear decrease in the fraction of circulating MAITs, corroborating an earlier report with a smaller sample [15]. Furthermore, our extended characterization of MAITs reveals that especially the CD8^+^ subset was reduced, while the remaining MAIT cells were more often activated and retained their ability to produce IFN‐γ.

### Proportion of MAITs does not correlate with anti‐commensal antibodies

**Figure 2:**
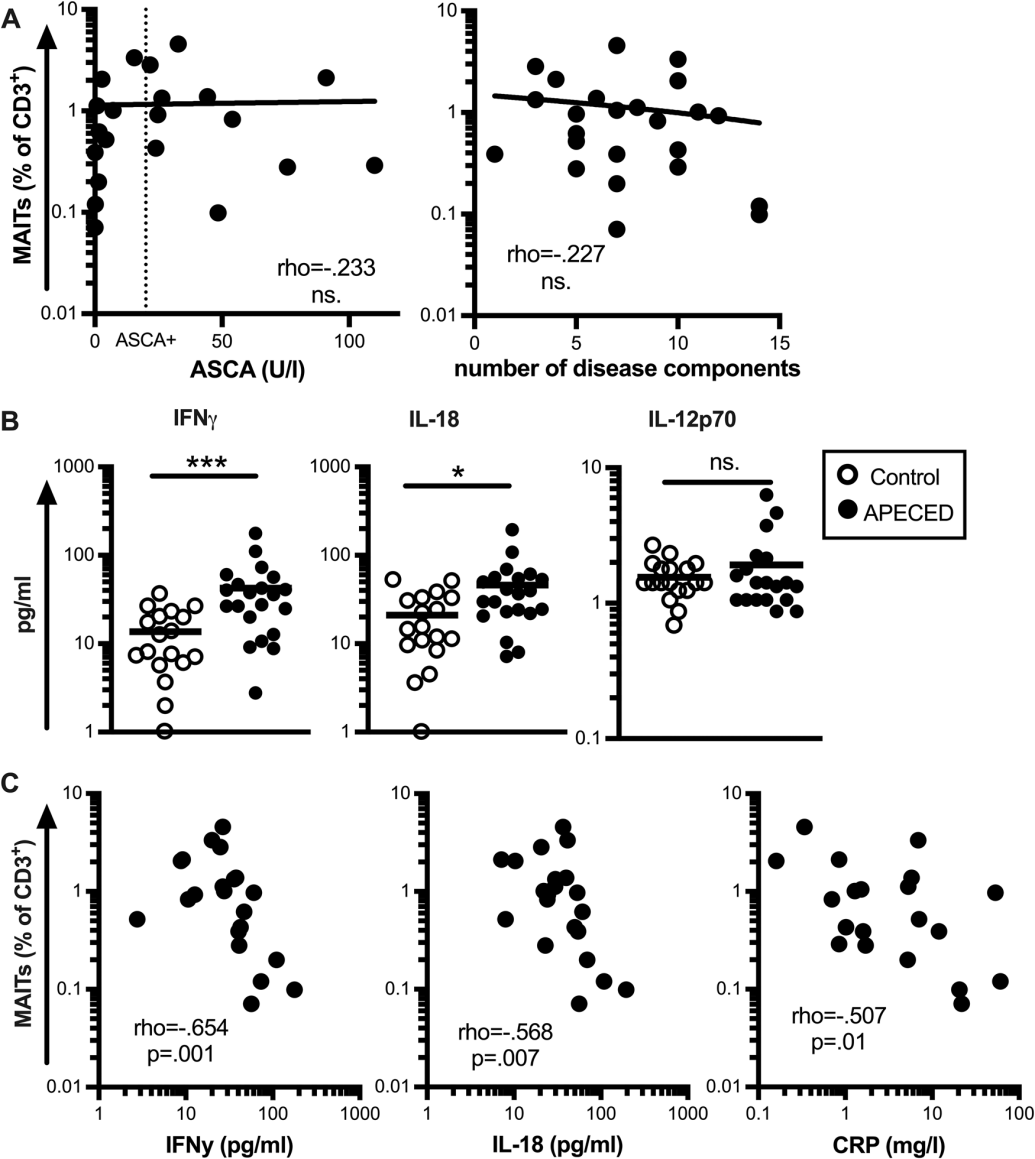
Association of MAIT proportion to serum cytokine concentrations in patients with APECED. (A) Correlation of anti‐*Saccharomyces cerevisiae* antibodies (ASCA) and number of disease components with the proportion of circulating MAITs in patients with APECED. (B) Serum IFN‐γ, IL‐18, and IL‐12 concentrations in controls (open circle) and patients with APECED (black). (C) Correlation of serum IFN‐γ, IL‐18, and CRP concentrations with the frequency of MAITs in patients with APECED. Statistical significance was calculated with nonparametric Spearman's rho (A, C) and Mann–Whitney *U*‐test (B); *p < 0.05, ***p < 0.001, ns. = non‐significant.

MAIT homeostasis is intimately influenced by interactions with commensal microbiota. We have previously shown that patients with APECED have altered gut microbiota characterized by expanded *Bacteroidetes* and *Proteobacteria* phyla, which include many microbes encoding the riboflavin pathway capable of activating MAITs [19, 20]. APECED patients also have increased antibody responses against commensal microbes [21], and chronic gut inflammation in APECED could account for increased sequestration of MAITs into the gut mucosa as is observed in inflammatory bowel disease [22]. We next studied whether altered anti‐commensal immunity might be associated with the reduction of MAITs using anti‐*Saccharomyces cerevisiae* antibodies (ASCA) as a marker. In Crohn's disease, ASCA antibodies are associated with more severe disease [23]. Twelve out of the 21 (57.1%) tested patients were positive for ASCA compared with 4 out of 22 controls (18.2%; Fisher exact *t*‐test *p* = 0.01). However, no correlation between ASCA and MAIT frequency was observed, nor did we see any difference in the frequency of MAITs in ASCA‐positive and negative patients (Fig. 2A, Supporting Information Fig. S4A).

### Frequency of MAITs does not align with the number of disease components

We also tested for possible correlation between the frequency of MAITs and the severity of APECED. There are currently no established scoring criteria or laboratory markers to measure the clinical severity of APECED. Therefore, as a surrogate, we used the number of disease components as a robust proxy, but no correlation was observed (Fig. 2A). Further, the frequency of MAITs had no correlation with patient age (Spearman's rho −.206, ns.).

### Reduced proportion of circulating MAITs does not correlate with anti‐cytokine antibody levels

A previous publication examining MAITs in APECED hypothesized that their reduction might be associated with neutralizing anti‐cytokine antibodies as neutralizing IL‐22 antibodies were also found in the patients’ saliva [15]. Neutralizing antibodies against IL‐22 were detected in 23/24 of our patients. With such a high rate of patients displaying neutralizing antibodies, we were unable to make comparison to patients without antibodies, but the titer of neutralizing anti‐IL‐22 antibodies did not correlate with the frequency of MAITs in patients (Supporting Information Fig. S4B). Another possible cause of reduced MAITs might be antibodies against IL‐12/23, reported to cause an MAIT defect in patients with thymoma, a neoplasm of the thymus with somatically reduced expression of AIRE [24]. However, no anti‐IL12/23 antibodies were detected in our cohort of patients with APECED (data not shown).

### MAIT proportion shows a clear negative association with serum IFN‐γ, IL‐18, and CRP concentration

MAITs are activated upon stimulation with IL‐18 and IL‐12 [11, 13]. We therefore measured the concentration of these cytokines in the serum using a flow‐cytometry‐based multiplexed assay. The IL‐18 concentration was significantly elevated in patients compared with healthy controls (42.6 vs. 13.7 pg/mL; *p* < 0.001; Fig. 2B). The concentration of IL‐12 was elevated in three patients but its overall level was comparable in patients and controls (1.7 vs. 1.5 pg/mL; ns.; Fig. 2B). We also found serum IFN‐γ to be increased in patients (46.0 vs. 21.1 pg/mL; *p* = 0.01) supporting previous findings of a pronounced Th1 skewing in patients’ circulating T lymphocytes and mucosa [7, 8, 15]. Using the acute phase protein C‐reactive protein (CRP), a widely used clinical marker of inflammation, we detected low‐grade inflammation in many of the patients (range 0–61, mean 7.78 mg/L; Fig. 2C). The serum concentrations of IL‐18, IFN‐γ, and CRP all showed a clear negative correlation with the proportion of circulating MAITs (Fig. 2C).

Reduction of circulating MAITs is not an uncommon finding in IEI, but a causal link between the genetic defect and MAIT reduction, except for a few specific genetic defects, has not been established [16]. Depletion of circulating MAITs has also been reported in numerous other conditions ranging from acute bacterial to chronic viral infections, autoimmune disease, cancer, gout, and even repeated injuries [12, 14, 25–30]. What these conditions of greatly varying etiology have in common is inflammation. MAITs do not need a specific antigen for their activation but can also be activated by inflammatory cytokines alone. They have a high expression of IL‐18 receptor and sensitivity for IL‐18 stimulation. In multiple sclerosis and systemic lupus erythematosus, reduced frequency of circulating MAITs correlates with serum IL‐18 concentration [25, 26]. In systemic lupus erythematosus, freshly isolated MAITs were prone to apoptosis [25], and prolonged exposure to IL‐12 and IL‐18 led to their death in vitro [12]. While we did not have a chance to study if IFN‐γ or IL‐18 stimulation directly leads to cell death of MAITs in APECED patients, given the negative correlation we observe in APECED between the frequency of circulating MAITs with serum IL‐18 as well as IFN‐γ concentration, we propose that the reduction in the proportion of circulating MAITs is secondary to their excessive consumption in chronic type 1 inflammation milieu.

### Data limitations and perspectives

We could not study the thymic development of MAITs in APECED, but we deem it unlikely that it would be disturbed, as MAIT commitment in the thymus relies on MR1‐dependent recognition of predominantly commensal antigens presented by cortical CD4^+^CD8^+^ thymocytes [31]. MAIT commitment thus occurs in the thymic cortex where AIRE is not expressed [32], although indirect effects in the thymic microenvironment caused by AIRE deficiency cannot be ruled out.

While analyses of chemokine receptor expression indicated no difference in MAITs of the patients and controls, without biopsies we cannot exclude the possibility that MAIT reduction in the circulation might reflect their increased homing to the gut or other tissues. Identifying MAITs in formalin‐fixed paraffin‐embedded tissue samples is currently not possible, the development of such a method would advance our understanding of MAITs’ distribution in human tissues.

## Conclusion

In conclusion, we establish a strong association between excessive type I inflammation and a reduction in the proportion of circulating MAITs in patients with APECED without observing a link with anti‐cytokine antibodies. A similar phenomenon is likely at play in other IEIs characterized by overt immune activation, which is in many conditions IFN‐γ driven [33]. Although the root causes of excessive inflammation in different conditions may vary, the innate‐like nature of MAITs might render them vulnerable to the detrimental effects of acute or chronic immune dysregulation. For instance, a reduced frequency of MAITs is observed during the acute inflammation phase in Puumala virus and SARS‐CoV2 infection [29, 30]. Interestingly, after a hantavirus infection, the MAIT cell compartment is completely restored, while after severe COVID‐19, MAITs recover with signs of residual functional abnormalities [29, 30]. It remains intriguing to see whether the treatment of chronic type I inflammation in APECED with JAK inhibitors [34] will lead to full restoration of the MAIT compartment and improved mucosal integrity. We also advise caution in interpreting the reduction in MAIT frequency as a “disease‐specific” feature as it is often likely also in other conditions the consequence of excessive overall inflammation.

## Materials and methods

### Study cohort

The recruitment of patients and collection of clinical data have been previously described [3]. PBMCs were available from 24 patients with APECED with a mean age of 40.4 years (age range 7–70; 13 women). The following clinical components were assessed when calculating the number of disease components: adrenal insufficiency, hypoparathyroidism, candidiasis, alopecia, enamel dysplasia, oral squamous cell carcinoma, keratitis, vitiligo, rash with fever, hypogonadism, type I diabetes, diarrhea, obstipation, gastritis, celiac disease, malabsorption, hepatitis, exocrine pancreatic insufficiency, asplenia, nephritis, hypothyroidism, growth hormone deficiency. The control group consisted of 26 healthy volunteers (mean age 38.2 years, age range 10–66, 15 women).

The study was conducted according to the principles of the Declaration of Helsinki. The study was approved by the Ethics Committee of Helsinki University Hospital. All study participants or their guardians (for subjects aged <18 years) gave informed written consent.

### Flow cytometry

PBMCs were isolated using Ficoll‐Paque (GE Lifesciences) gradient centrifugation. The cells were frozen fresh after the isolation with CTL‐Cryo ABC (CTL) freezing kit and stored at −140°C before analysis. Fresh or thawed cells were incubated for 30 min at +4°C with antibodies and with live/dead fixable green dead cell stain (at dilution of 1:500; ThermoFisher) that were diluted in Brilliant Stain Buffer (BD Biosciences) for staining of surface antigens. For detection of transcription factors, the cells were permeabilized after surface staining with FoxP3 transcription factor staining set (eBioscience) and of intracellular cytokines with fixation/permeabilization solution kit (BD Bioscience) as instructed by the manufacturer. The data was acquired using LSR Fortessa (BD Biosciences) and analyzed with FlowJo (BD Biosciences, LLC). The gating was mostly done using biological negative populations. The antibodies used in the study are shown in Supporting Information Table S1. The optimal concentration for antibodies was titrated with live PBMCs.

For analysis of chemokine receptor expression thawed cells were stained with Fixable Viability Stain 440UV (at dilution of 1:1000, BD Bioscience) for 10 min at room temperature. A cocktail containing antibodies for chemokines receptors and MR1 tetramer in a mix of Brilliant Stain Buffer (BD Bioscience) and True‐Stain monocyte blocker (BioLegend) with Human TruStain FcX (BioLegend) was added and incubated first at room temperature for 10 min, followed by at 37°C for 40 min. After washing, antibodies for surface antigens were added for staining at 4°C for 6 h. Permeabilization was performed as mentioned above. Intracellular staining was carried out at 4°C for 16 h. The data was acquired using FACSDiscover S8 cell sorter (BD Biosciences).

### MAIT stimulation

*C. albicans* were fixed with CellFIX (BD Biosciences) and added to the cell culture in a concentration of 3 × 10^6^ CFU and an unconjugated anti‐CD28 antibody was added after 1 h of culture. Cells were incubated for 18 h and Brefeldin A (BD Biosciences) was added for the last 6 h. For PMA stimulation a commercial PMA/ionomycin preparation was used (Leukocyte Activation Cocktail, with BD GolgiPlug, BD Biosciences) for 6 h.

### Cytokine, CRP, and anti‐*Saccharomyces cerevisiae* antibody quantification

Cytokine 25‐Plex Human ProcartaPlex Panel 1B (Thermo Fischer) was performed from serum samples stored at −80°C. The results were acquired with the Bio‐Plex 200 system (Biorad)

CRP was measured from all patients and hsCRP was measured from all but four patients (*n* = 20) in a clinically validated laboratory at HUS Diagnostic Center (Helsinki, Finland). These data were combined for the correlation analysis. The four patients from whom we did not have hsCRP measurement available all had CRP under the detection rate (<3) with the less sensitive method and were excluded from the correlation analysis.

Antibodies against *S. cerevisiae* were evaluated with anti‐*Saccharomyces cerevisiae* antibodies IgG test kit (Bio‐Rad) and the cutoff for positive samples was set at 15 U/mL as instructed by the manufacturer. Absorbances were read using the iEMS reader MF (Labsystems).

### Anti‐cytokine antibody measurements

The anti‐cytokine antibodies were measured using a radioligand binding assay as previously described [3].

### Statistical analysis

The statistical analysis was performed using SPSS version 23 (IBM). Nonparametric Mann–Whitney *U*‐test and Spearman's rank correlation coefficient were used with the limit for statistical significance set to 0.05.

## Conflict of interest

The authors declare no financial or commercial conflict of interest.

## Author contributions

All authors contributed to the study's conception and design. Sample collections were performed by Saila Laakso and Outi Mäkitie, and experimental preparation and data analysis were performed by Iivo Hetemäki, Joona Sarkkinen, Huai Hui Wong, Nelli Heikkilä, Simo Miettinen, and Mikko Mäyränpää. The first draft of the manuscript was written by Iivo Hetemäki, and all authors commented on previous versions of the manuscript. All authors read and approved the final manuscript.

## Ethics approval statement

The study was conducted according to the principles of the Declaration of Helsinki. The study was approved by the Ethics Committee of Helsinki University Hospital.

## Patient consent statement

All study participants or their guardians (for subjects aged <18 years) gave informed written consent.

### Peer review

The peer review history for this article is available at https://publons.com/publon/10.1002/eji.202451189.

**Abbreviations**
APECED: autoimmune polyendocrinopathy candidiasis ectodermal dystrophy
AIRE: autoimmune regulator
CRP: protein C‐reactive protein
DN: double negative
IEI: inborn errors of immunity
MAIT: mucosal‐associated invariant T cell
5‐OP‐RU: 5‐(2‐oxopropylideneamino)‐6‐d‐ribitylaminouracil

## Supporting information



Supporting Information

Supporting Information

## Data Availability

The datasets generated during and/or analyzed during the current study are available from the corresponding author upon reasonable request due to privacy restrictions.
