# Supplementary material for: Reduction in mucosal‐associated invariant T cells (MAIT) in APECED patients is associated with elevated serum IFN‐γ concentration
Source: Eur J Immunol. 2024 Sep 18;54(12):2451189. doi: 10.1002/eji.202451189 (PMC11628920; doi:10.1002/eji.202451189)
Supplement: Supplementary file 1 — Supporting Information [file EJI-54-2451189-s001.pdf]

Suppl Fig 1

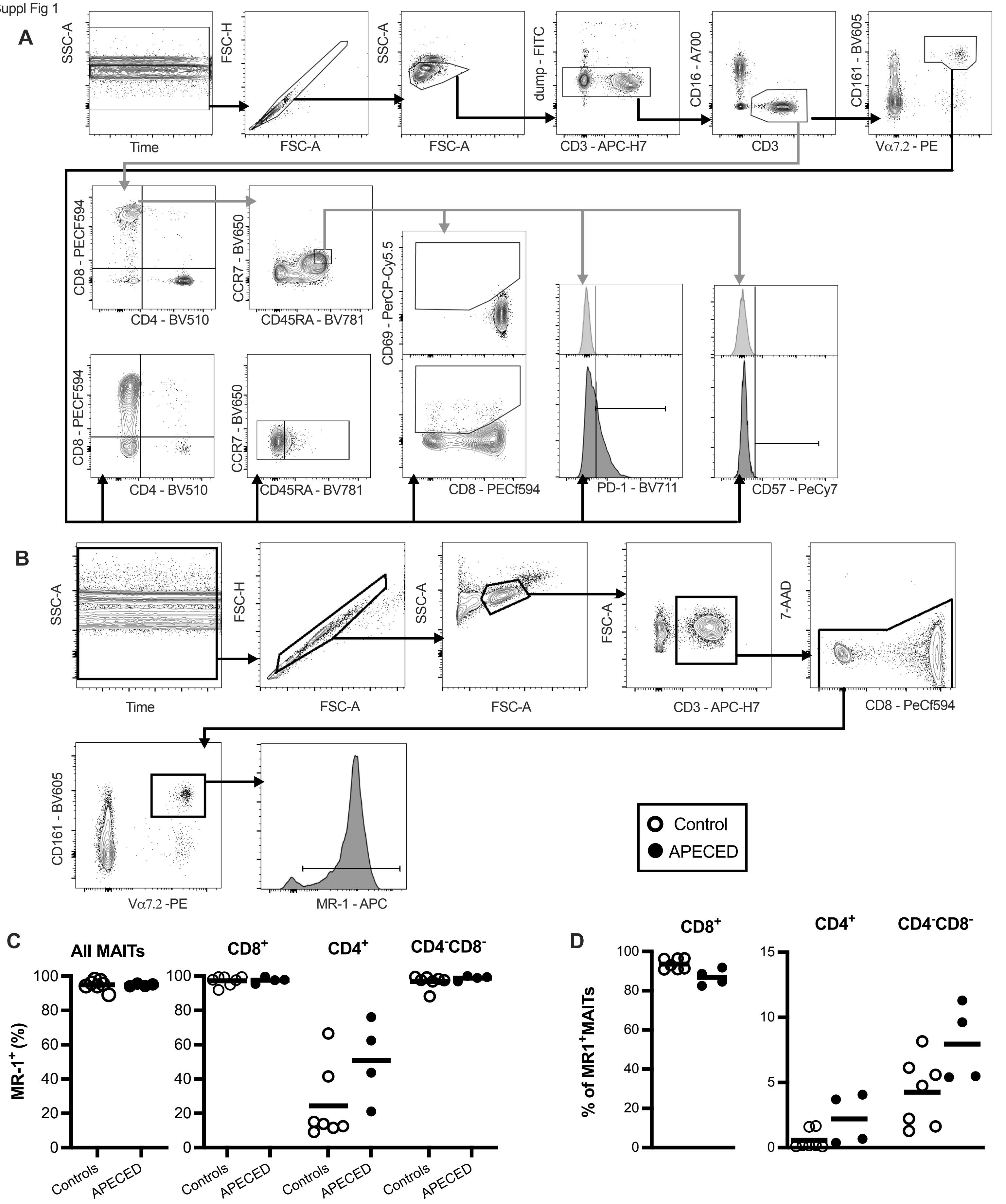

Supplementary Figure 1 - Gating strategy for Figure 1A-D

A) Gating strategy to identify MAITs from peripheral blood and analyse their maturation and activation status. Vα7.2^{+}CD161^{++} MAITs were identified from live CD14^{-}CD19^{-}CD16^{-}CD3^{+} T cells. The CD8^{+}CD4^{-} and CD8^{-}CD4^{+} and CD45RA^{+} population were analysed. CD69^{+}, PD1^{+} and CD57^{+} populations were gated with the help of expression of said markers in CD4^{-}CD8^{+}CCR7^{+}CD45RA^{++} population. Gating strategy is shown in a healthy 24-year-old male. B) Gating strategy to identify MR-1 expression in freshly isolated MAITs. Vα7.2^{+}CD161^{++} MAITs were identified from live CD3^{+} T cells and cells binding to MR1-tetramer loaded with 5-(2-oxopropylideneamino)-6-D-ribitylaminouracil (5-OP-RU) were gated from them. Gating strategy is shown in 44-year-old female. C) MR-1 binding of all Vα7.2^{+}CD161^{++} MAITs, CD8^{+}CD4^{-}, CD4^{+}CD8^{-} and CD4^{-}CD8^{-} MAITs, respectively, in healthy controls (n=7, open circle) and patients (n=4 black). D) Frequency of CD8^{+}CD4-, CD4+CD8-, and CD4-CD8- cells of all Vα7.2^{+}CD161^{++}MR1^{+} MAITs. Dump = exclusion channel for dead cell marker, CD19, and CD14.

Suppl Fig 2

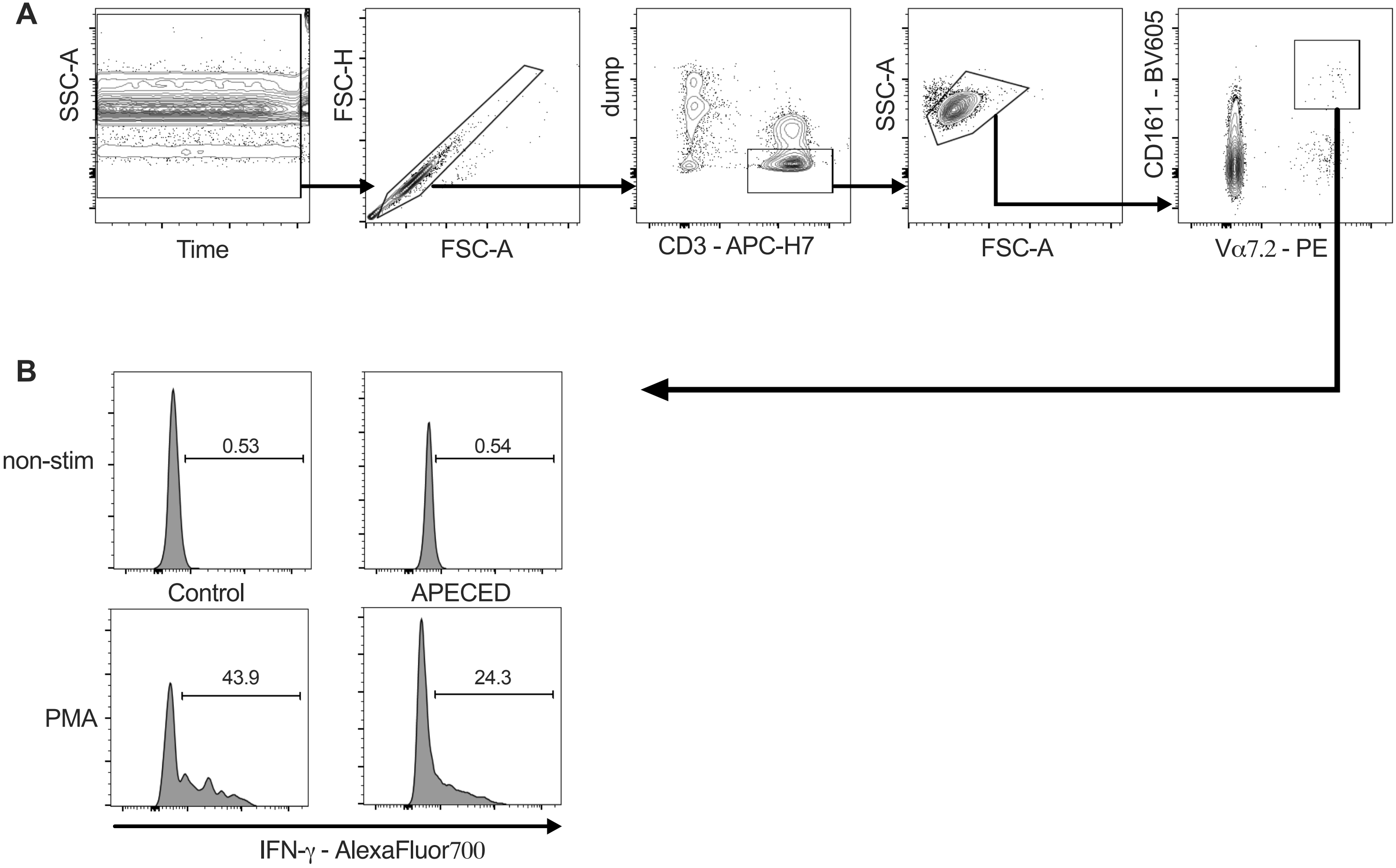

Supplementary Figure 2 - Gating strategy for 1E & F

A) Gating strategy to identify MAITs and B) assess IFN-γ expression in them. IFN-γ expression in MAITs without and with PMA stimulation in representative age-sex matched control and patient with APECED is shown. Dump = exclusion channel for dead cell marker, CD19, and CD14.

Suppl Fig 3

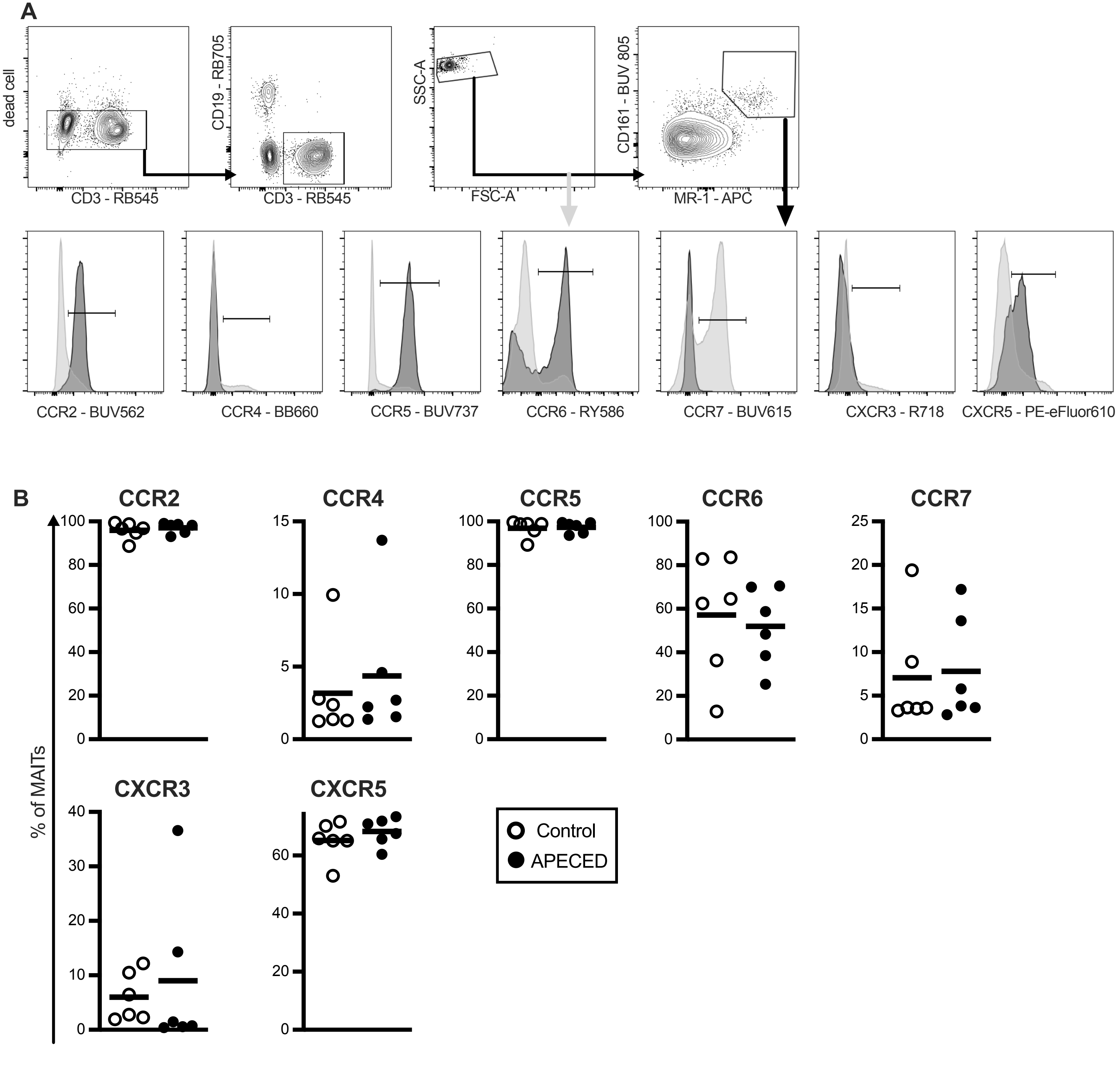

Supplementary Figure 3 - Chemokine receptor expression in MAITs

A) Gating strategy to identify MAITs from peripheral blood and analyse their chemokine expression. Single cells were gated as in Suppl Figure 1A and from them MR1$^+$CD161^{++} MAITs were identified from live CD19$^-$CD3$^+$ T cells. MR1-tetramer was loaded with 5-(2-oxopropylideneamino)-6-D-ribitylaminouracil (5-OP-RU). Chemokine receptor expression was gated from MAITs. Expression of chemokines in all CD3+ (grey) and in MR1$^+$CD161^{++} MAITs is shown (black). Gating strategy is shown in a healthy 66-year-old female.

B) Expression of CCR2, CCR4, CCR5, CCR6, CCR7, CXCR3 and CXCR5 in healhty controls (open circle) and patients with APECED (black).

Suppl Fig 4

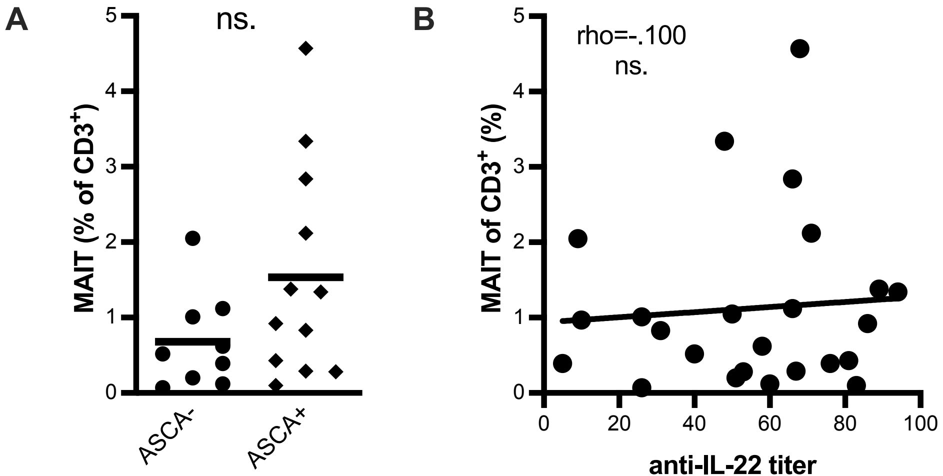

Supplementary Figure 4 – Association of MAIT frequency to patient characteristics

A) Frequency of MAITs in patients with APECED without or with anti- *Saccharomyces cerevisiae* antibodies (ASCA). B) Correlation between anti-IL-22 antibody titer and frequency of MAITs in patients with APECED. Statistical significance was calculated with Mann-Whitney U-test (A) and Spearman’s rank correlation (B).
